# Democratic Justifications for Patient Public Involvement and Engagement in Health Research: An Exploration of the Theoretical Debates and Practical Challenges

**DOI:** 10.1093/jmp/jhad024

**Published:** 2023-05-25

**Authors:** Lucy Frith

**Affiliations:** Centre for Social Ethics and Policy School of Law, The University of Manchester, Manchester, UK

**Keywords:** deliberation, democracy, democratic theory, health research, participation, patient public involvement and engagement

## Abstract

The literature on patient public involvement and engagement (PPIE) in health research has grown significantly in the last decade, with a diverse range of definitions and topologies promulgated. This has led to disputes over what the central functions and purpose of PPIE in health research is, and this in turn makes it difficult to assess and evaluate PPIE in practice. This paper argues that the most important function of PPIE is the attempt to make health research more democratic. Bringing this function to the fore and locating PPIE in the wider context of changes in contemporary forms of democratic engagement provides greater conceptual clarity over what PPIE in research should be trying to achieve. Conceptualizing PPIE as a form of democratization has a number of benefits. First, theories of what are appropriate, normatively justifiable and workable criteria for PPIE practices can be developed, and this can provide tools to address the legitimacy and accountability questions that have troubled the PPIE community. Second, this work can be used to form the basis of a research agenda to investigate how PPIE in health research operates, and how it can facilitate and/or improve democratic processes in health research.

## I. BACKGROUND AND DEFINITIONS

The institutionalization of patient public involvement and engagement (PPIE) in health research is a relatively recent development. The drive for more PPIE has come from a range of different groups, from activists and patient groups, professional organizations to government bodies, and has been widely endorsed and promoted at both national and local policy levels. In the United States, initiatives such as the Patient-Centered Outcomes Research Institute (PCORI) require all grants to have an “engagement plan” for how they are going to involve the public. The Canadian Strategy for Patient-Oriented Research aims to engage patients as partners and encourage greater focus on patient-identified priorities (CIHR, 2014). In the United Kingdom, The National Institute of Health Research (NIHR), from its launch in 2006, has had a firm commitment to PPIE in the research process.

This paper focuses on PPIE in applied health research. This includes clinical research, clinical trials and studies on treatments and interventions, health service research, and work on the structures and organization of health and social care systems. This paper examines research practice itself, rather than policy-level decision-making, because there has been a substantial body of literature on PPIE at this level (see Abelson et al., 2007, for example) and less consideration of how PPIE is carried out in research programs and projects. While the arguments here could be applied to other kinds of health-related research, such as basic science studies, for clarity the focus is on the applied end of the research spectrum.

A large number of different terms are used to capture some form of “lay” role in health research, “patient representative,” “public advisor,” “public member,” “public contributor,” “consumer,” and “citizen.” These are often used interchangeably, and there is little clarity about whether these terms refer to distinct entities or are different names for the same entity. There are also a large number of terms used for PPIE, “citizen engagement,” “public participation,” and “community engagement,” all incorporating different nuances and assumptions (Fredriksson and Tritter, 2017). For the purposes of this paper, the term “patient public involvement and engagement” will be used to cover activities in which patients and members of the public take part that are designed to provide lay input into health research. “Public contributor” will be used to designate the lay or non-professional person who is there to provide the patient and/or public perspective.

Despite the wide endorsement of PPIE in health research internationally, there is little consensus over how PPIE should be defined. This is partly because PPIE covers a wide range of activities, and what constitutes involvement and engagement is contested. Boote et al. define it as: “Consumer involvement in research relates to an active relationship between consumers and researchers in the research process” (Boote et al., 2002, 214). INVOLVE, a UK organization dedicated to PPIE in health research,^1^ defines PPIE as “research being carried out ‘with’ or ‘by’ contributors of the public rather than ‘to’, ‘about’, or ‘for’ them.” (INVOLVE, 2019) Here, PPIE includes notions of active contribution and “good” PPIE is more about co-production than just involvement. A number of authors have attempted to develop categorizations of an “ideal type” of PPIE (Fredriksson and Tritter, 2017) and develop topologies to capture what PPIE is (Oliver et al., 2008; Tritter, 2009). Adernstein’s ladder of engagement is often quoted as one of the first examples of this. Involvement and engagement are seen as a continuum starting on the bottom rung of the ladder with manipulation and ending on the top rung with citizens’ power (Adernstein, 1969).

Authors have also tried to categorize PPIE based on the different roles the public contributor could potentially play and the differing relationships and power dynamics that these roles manifest (Williamson, 2014). One distinction that can be made is between patient involvement, the patients being involved in their own care, and public involvement, a member of the public or a citizen being involved in wider policy decisions (Charles and DeMaio, 1993; Fredriksson and Tritter, 2017; McCoy et al., 2019). As noted by Forbat et al. (2009) the role of the public contributor in research is a “distinct conceptualisation” that sits somewhere between being a patient and being a member of the public. Public contributors are often involved in research projects in their capacity as someone who has experienced the condition under investigation or has a particular life experience, not as a patient undergoing treatment or a general member of the public. The proliferation of definitions and topologies of PPIE makes it hard to determine the normative basis of PPIE (McCoy et al., 2018) and, in turn, has made it difficult to assess and evaluate PPIE practice (Mockford et al., 2012; Hughes and Duffy, 2018).

These definitional and terminological problems demonstrate a need for further analysis of the theoretical underpinnings of PPIE in health research. This paper argues that the most important function of PPIE is that it attempts to make health research more democratic. Bringing this function to the fore and locating PPIE in the wider context of changes in contemporary forms of democratic engagement provides greater conceptual clarity over what PPIE in research should be trying to achieve. Conceptualizing PPIE in this way, as a form of democratization, has a number of benefits. First, theories of what are appropriate, normatively justifiable and workable criteria for PPIE practices can be developed, and this can provide the tools to address the legitimacy and accountability questions that have troubled the PPIE community. Second, this work can be used to form the basis of a research agenda to investigate how well PPIE in health research functions as a democratic process, how it can be used to democratize research, and to provide a firmer basis for assessment of how PPIE practices meet these ends.

Democratization is used here to mean the ways that democracy in health research is furthered or promoted. As has been extensively noted, democracy is a contested concept (Kurki, 2010). Christiano (2015) defines democracy as: “a method of group decision-making characterized by a kind of equality among the participants at an essential stage of the collective decision-making.” (Christiano, 2015). Archiebugi and Held (2011) define democracy to mean the various ways the public can interact, be involved in, and influence decisions and practices. These definitions draw attention to different elements of the democratic process; deliberation, participation, and decision-making, and different theories privilege and draw attention to these aspects to varying degrees (Floridia, 2013). While precisely how democracy is defined depends on the value placed on these different aspects (Elstub, 2018), broad notions of involvement and influence underpin all conceptions of democracy.

## II. PPIE AS A FORM OF DEMOCRATISATION

This paper advances the argument that a key function of PPIE is the attempt to make health research more democratic. This function is central to many justifications of PPIE and has the potential to radically change health research practice. This is not the only justification for PPIE, but it is an extremely significant one and therefore deserves more focused attention. A number of justifications for PPIE can be found in the health research literature. It can: improve the quality of health care; make research more relevant to users; make the decision-making process more inclusive; and empower patients and the public (Gradinger et al., 2013). The accounts that explicitly consider the underpinning theoretical justification of PPIE generally divide them into two broad categories: promoting intrinsic values that should underpin health research and health care more generally (i.e., equality, trust, and transparency) and instrumental values (i.e., improving research and consequently health outcomes and reducing inequalities) (Beresford, 2007). Ives et al. (2013) draw attention to PPIE being both a means to an end and an end in itself. Endelman and Barron also make this distinction: PPIE can improve the quality and relevance of research, a consequentialist justification, but it could also give a “rightful voice and power to those at the heart of health care,” a deontological justification (2016, 209). Intrinsic justifications for PPIE seem self-evidently to embody a form of democratic commitment, and I argue that instrumental justifications can also contain elements of this type of commitment.

A key instrumental justification of PPIE is that the public contributor brings some kind of expertise or new form of knowledge to the research process. “The contributions of patients can be extremely valuable, providing alternative views from those of the research team or [research] staff. Patients are able to make judgements based on their understanding and lived experience of their condition.” (RDS Handbook, 2018) This is often seen as a form of expertise by experience. For example, the public contributor has experienced the condition that is being researched, hence, they can give a different perspective on determining the areas that need to be investigated and provide a patient-centered view of what a good outcome might look like (Solomon et al., 2016; Largent et al., 2018). It is argued that bringing these different lay perspectives can lead to improvements in the evidence base of health care by designing research that matches what users want and need and ensuring that research is relevant to patients. Hence, the outputs of the research are more likely to be adopted and used in practice (Dudley et al., 2015).

This bringing of expertise and the democratic impetus of PPIE are often put on either side of an instrumental versus intrinsic justification for PPIE in research. However, as Martin (2008) points out, although the literature sets up two different types of justification for PPIE—a democratic, intrinsic, justification, that the public should have a say in how health research is carried out; and a technocratic, instrumental, justification, that different types of expertise are needed that only the public can provide—there is not always a clear distinction between these two justifications. The role of contributing a different form of expertise to the research process is not just part of bringing more information to bear on a research problem, it can also be premised on some form of democratic commitment. The expertise of public contributors brings epistemic diversity to the research process. This widens the epistemic terrain so that more and varied viewpoints can be included.

A recent movement within the field of democratic theory focuss on what are termed instrumental justifications of democracy so that, as well as fostering just and fair procedures, it can produce better outcomes. This “epistemic turn” has been advanced by theorists such as Landemore, Estlund (2007), and Goodin (2007). “It rehabilitates instrumental, consequentialist, and ‘best outcomes’ arguments for democracy and makes it possible to base the value of democracy as a whole, and the legitimacy of its procedures, at least partly on its ability to generate good outcomes.” (Landemore, 2017, 290) Thus, what are often termed purely instrumental reasons for PPIE also can be seen to include an epistemic justification for democracy, where the gathering of more information, opinions, and knowledge that democratic processes foster, leads to better decisions. Hence, the democratizing element of PPIE is not only key to intrinsic justifications of PPIE, but an important element of particular knowledge bringing instrumental justifications for PPIE as well. This is not to say that all instrumental justifications incorporate this commitment; often PPIE fulfills an information-bringing function without any attempt democratically to engage public contributors. Much of current PPIE practice in health research operates in a limited way, where public contributors comment on already-formed proposals. This limits its democratic effects because it does not give public contributors the opportunity to really influence the research process.

However, if PPIE in practice is able to operate in a way that enables genuine involvement and co-production, it can be a useful mechanism for democratizing health research. This is an important and key function of PPIE, because it has the potential to change health research both in terms of what is studied and how research projects and programs are organized. It can broaden conceptions of what is counted as “scientific” knowledge and create greater equality in knowledge production—rather than concentrating it in the hands of an elite (Hutchinson et al., 2017). PPIE is not just a way of getting data on the public’s views or patients’ preferences, data that could be gathered more comprehensively and efficiently, for example, through large-scale surveys, or explored more extensively by in-depth qualitative work. Public contributors are more than research participants and subjects, they are more than a way of generating data or knowledge on a specific topic. Involving public contributors in the knowledge production process, through co-production, and more democratic forms of involvement, could prompt a reorientation of applied health research and be a way of challenging and rethinking research practice. This could produce subsequent health benefits, particularly for marginalized communities, by improving health outcomes and reducing health inequalities. These are communities that are often under-represented in research studies, prioritization processes, and the wider governance and oversight of research.^2^ This radical potential can sometimes get lost in debates over justifications for PPIE; the purpose of this paper is to foreground this conceptualization, PPIE as a way of democratizing research, so that PPIE’s radical potential can be more fully realized.

Democratizing research is something distinctive that PPIE can bring to the research process. In theory, there could be other ways of democratizing health research; for example, all citizens could vote in mini-health referendums. However, these would be difficult and expensive to organize, and such referendums are more suitable to bigger policy issues, such as the referendum held in Ireland on whether to remove the constitutional ban on abortion in 2018. In sum, it has been claimed that one of the most important functions of PPIE in research is to democratize the research process. Other driving forces for PPIE arguably run counter to this. For example, the consumerist and individualistic drivers for such involvement (Gibson et al., 2012) and “the wider politics of knowledge in which patient groups, clinicians and universities are co-opted into a [neo-liberal] corporatized health research agenda” (Madden and Speed, 2017, 5). Further, not all justifications and functions of PPIE can be cashed out in terms of their democratizing capabilities. Nevertheless, the democratizing function of PPIE is important because it has the potential to radically transform how research is practiced.

## III. PPIE AND WIDER TRENDS IN DEMOCRATIC ENGAGEMENT

Having argued that making health research more democratic is the most important function of PPIE in applied health research, I will now consider how PPIE relates to new forms of democratic engagement. PPIE can be seen as part of the trend of moving engagement beyond traditional democratic structures. Locating it in these wider trends can elucidate how PPIE functions as a mechanism of democratization and can provide theoretical tools to open up new ways of conceptualizing PPIE in health research.

Attempts to widen democratic engagement beyond the ballot box have grown in recent years. It is widely agreed that there is a crisis in electoral representative democracy: electoral turnouts are decreasing and mainstream politics is suffering a legitimation crisis (Fishkin and Mansbridge, 2017). Yet, people still want to be involved in public decision-making; this is now taking different forms, such as citizen juries and people’s parliaments (Fung, 2003). Alongside this, there is a growing theoretical focus on the idea that democracy is any arrangement that enables all those affected by a decision to be able to influence it—“democratic autonomy”—and this can take place outside the conventional forms of electoral representation and its associated institutions (Urbinati and Warren, 2008).

In response to the crisis in representative democracy, new forms of democratic engagement have arisen, democratic innovations that change the way people can influence the polity (Smith, 2009; Elstub and Escobar, 2017). According to Elstub and Escobar (2017), what binds all these concepts of democratic innovation together in a “Wittgensteinian ‘family’ of conceptual clusters” is that “they all reimagine the role of citizens in governance processes, and thus renegotiate the relationship between government and civil society” (Elstub and Escobar, 2017). PPIE in research arguably constitutes a “new” approach to democratization, a form of democratic innovation; it is becoming an institutionalized way of involving citizens in forms of research governance and practice. Abelson et al. (2003, 2013) chart the rise in participatory models of decision-making in health care more broadly, observing that “An active, engaged citizen (rather than the passive recipient of information) is the prescription of the day” (2003, 240). This democratizing of health care has been justified on a number of grounds. Because health care involves spending “public money” generated from taxes, be that public insurance plans or direct provision depending on the health system, there is a view that the public should be involved in the oversight of this. Further, in any health system it is argued that public values and sentiments should be incorporated at meso and micro levels, rather than only at state or national policy levels. There is also a recognition that the problems facing modern healthcare systems are ethically and politically challenging, and therefore there is a need for collective problem-solving and incorporating public values into policy-making (Abelson et al., 2013). Good democratic processes in health care can arguably “further extend the amount and types of information incorporated into decisions, empower citizens to be pro-active in protecting their lives and health, and ensure that experts alone are not charged with making value-laden decisions” (Tickner, 2001, 94). PPIE in research can be seen as part of this broader trend; it rests on the premise that patients and the public should have a right to have a say in how health research is conducted and what is researched, a form of “no decision about me, without me” (Coulter and Collins, 2011). Thus, health care and health research are now seen as areas where some form of democratic decision-making is appropriate, with cultural expectations that the “public” is involved in these decisions in some way (Solomon and Abelson, 2012).

## IV. DEVELOPING NORMATIVE THEORIES

Conceptualizing PPIE in research as a way of democratizing health research clarifies what the most important function of PPIE *should be*. By locating PPIE as part of the changing face of modern democratic practice, theories of appropriate, normatively justifiable and workable criteria for PPIE in health research can be developed. As has been noted, democratic innovation can take many forms (Elstub and Escobar, 2017), and what form it takes in health research needs to be further elaborated. PPIE in health research consists of deliberative spaces where people come together to discuss health research, populated by unelected public contributors. Currently, the number of active participants in these processes is limited, which makes participatory models of democracy less relevant for exploring this area. Floridia sums up the differences between deliberative and participatory theories of democracy: “participatory democracy is founded on the direct action of citizens who exercise some power and decide issues affecting their lives; deliberative democracy, instead, is founded on argumentative exchanges, reciprocal reason giving, and on public debates which precede decisions” (Floridia, 2013, 6).^3^ The way that PPIE in research is currently carried out suggests that it can be most productively conceptualized as a form of deliberative rather than participatory democracy—deliberative forms of democracy most closely resemble how democratic procedures in PPIE in health research are carried out.^4^ Conceptualizing it in this way can provide useful theoretical tools to examine and assess PPIE in health research.

Deliberative democracy provides a rich resource for considering how PPIE operates in health research as a democratizing process. Kuyper notes “because these actors [public contributors] …articulate representational claims and mobilize constituencies through discursive practice, deliberative democracy offers a robust theoretical toolkit... to evaluate these agents” (Kuyper, 2016, 308). The process of deliberation, debate, and claim-making form the central features of the public contributors’ role. Abelson et al.’s (2003) “active citizen” who is engaged and participates in healthcare decision-making is involved in these discursive practices (Dryzek, 2002), taking on a number of quasi-representational roles and functions, functions that at their root have a commitment to democratic ideals (Blacksher, 2013).

Deliberative democracy has gained increasing popularity as a theory that considers democracy beyond the ballot box and is a key part of many of the new forms democratic engagement that are growing in contemporary society. As Dryzek notes: “The essence of democracy itself is now widely taken to be deliberation, as opposed to voting, interest aggregation, constitutional rights, or even self‐government” (2002, 1). Deliberative democracy can broadly be defined as: “Collective decision-making with the participation of all who will be affected by the decision or their representatives: this is the democratic part... It includes decision-making by means of arguments offered by and to participants... this is the deliberative part” (Elster, 1998, 8). Talk-centric democracy replaces voting-centric democracy, the communicative process is privileged, how matters are discussed and debated, and how opinion is changed as a result are key elements of deliberative democracy (Curato et al., 2017). A process is democratic if those involved have had the opportunity to deliberate about those decisions.^5^

Deliberative democracy can provide the tools to address legitimacy and accountability questions that have troubled those considering non-electoral representation (Montanaro, 2012) and PPIE in health research specifically (Maguire and Britten, 2017). Namely, how can PPIE in health research be said to be legitimate without such accountability measures provided by conventional electoral democratic processes? One of the key practical mechanisms of current democratization initiatives in health research is involving public contributors in research programs and projects. How public contributors act as representatives in these forums, what gives them legitimacy to make decisions, and how they are and should be held accountable, is now considered.

Public contributors play a specific form of representational role in health research, a form of non-electoral representation. As Urbinati and Warren note, these forums, be they citizens juries, panels, etc., are a growing phenomenon in contemporary social life. “The forums involve non-elected, formally designed venues... [where] a few citizens actively serve as representatives of other citizens” (Urbinati and Warren, 2008, 405). Public contributors are called on to speak, in some way, for their “constituents,” whether these are the wider public, people with a certain health condition, or people who have had particular experiences, such as being a caregiver. How public contributors can be said to speak on others’ behalf or “represent” “those who are not present” and even if they should be seen in this way has been widely debated in the literature (Maguire and Britten, 2017; Lander et al., 2019). It is frequently claimed that public contributors cannot really be representatives, because they cannot speak for all patients, or even for all patients with certain conditions. These are perennial problems of representation, a difficulty inherent in one person’s claiming to represent many people (as Iris Maron Young [1986] points out). However, it is claimed that additional problems beset public contributors in health research. First, it is often said that public contributors are insufficiently diverse in demographic characteristics and experience and therefore are not able to bring the views of the wider population to the research process (Baggott, 2005). This is potentially important, because bringing a diversity of viewpoints is one of the main justifications for PPIE; not achieving this could defeat the very goal of doing PPIE. Second, and less often mentioned in the health research literature, is the issue that public contributors have not canvassed the views of their wider community, nor been elected to take certain platforms forward, and therefore cannot act as a conduit between their community and researchers (Fischer and Van de Bovenkamp, 2019).

While these may be important issues that need to be considered in PPIE practice, the legitimacy of the representational role in PPIE does not solely rest on this type of claim. The representational role of PPIE in research is different from traditional political forms of electoral representation where the goal is to get participation from all sectors of society based on statistical conceptions of representation. We need to think of representation in PPIE in a more dynamic way and not see statistical representation as the gold standard. Instead, we should pay attention to the “range of pragmatic and productive connections [PPIE] engenders” (Martin, 2008, 49). The complexities of representation in modern society are being increasingly recognized: “there’s a lot ‘going on’ in representation, a constant process of making, receiving, accepting or rejecting representative claims” (Saward, 2008, 1004). Further, representation is always partial—we are never fully represented. These elements, along with the uncoupling of representation from statistical constructions, mean that representation takes on a different form in the context of health research. These forms are not static but processes of negotiation and claim-making. Representation can be seen “as a dynamic and productive practice in context, rather than a phenomenon restricted to a grid of preconceived and acontextual categories” (Saward, 2014, 725). How these contextual factors work to produce different types of representation needs to be further explored. Questions over the role of representatives and constructions of representation in PPIE in research are part of wider debates over concepts of representation that sit outside electoral institutions. With the growing number of forums made up of non-electoral representatives that are now becoming part of wider public decision-making processes (Urbani and Warren, 2008), such questions are becoming increasingly important for modern societies.

Using the tools of deliberative democracy, what gives decisions legitimacy in PPIE can be established: “A legitimate political order is one that could be justified to all those living under its laws... Accountability is primarily understood in terms of ‘giving an account’ of something, that is, publicly articulating, explaining, and most importantly justifying public policy” (Chambers, 2003, 311). Therefore, it is the deliberative processes by which decisions are made that are of central importance, and good deliberative processes will lead to more legitimate decisions and, according to some, more moral decisions. As Gutmann and Thompson argue, deliberative forums have a role in encouraging moral rather than self-interested decision-making; they quote John Stuart Mill, who points to the benefit of this for a citizen when “called upon... to weigh interests not his own; to be guided, in case of conflicting claims, by another rule than his private partialities; to apply, at every turn, principles and maxims which have for their reason of existence the common good” (2004, 149).

### Limits of Deliberative Democracy in PPIE

It has been claimed that conceptualizing PPIE in health research as a form of deliberative democracy can provide a way of developing theories of appropriate, normatively justifiable and workable criteria for PPIE in health research. There are two potential types of problem with this argument. First, there are theoretical objections to deliberative democracy: it sacrifices justice on the altar of deliberation; it tells us nothing about the legitimacy of a decision (Raz’s critique); it presupposes that we all agree what a good reason might look like and what counts as a good reason in particular situations; and problems with consensus forms of decision-making. There is not space in this paper to provide a full defense of deliberative democracy.^6^ How these critiques specifically relate to PPIE in health research, which ones have purchase, and how PPIE in research functions as a form of deliberative democracy and what limitations there are with this model are areas that the research agenda outlined below could address.

The second type of objection is that it is an impractical suggestion. It is not what currently happens in practice, and, further, it is unlikely to ever happen in practice, so deliberative democracy is not an appropriate model for democratizing health research. The notion of equals debating and opinions being changed by the deliberative process may seem far away from the reality of the way applied health research projects operate (Green, 2016). Deliberative democracy is often accused of being elitist: only those who are well educated and articulate can contribute to deliberative processes. As is noted in many different contexts, “inequality is always in the room” (Lupia and Norton, 2017); power and exclusion operate in a myriad of ways (Ocloo and Mathews, 2016). Further, PPIE as a deliberative practice is hard and requires substantial investment in time and resources, and, as authors have highlighted, some might see this as too exacting an enterprise to be carried out in everyday research practice (Abelson et al., 2003). Hence, there could be significant practical challenges to operationalizing deliberative democracy in health research. PPIE is a relatively recent development; consequently, such deliberative processes are arguably at a nascent stage. However, this does not detract from the usefulness of conceptualizing it in this way. Mansbridge et al. (2010, 65) draw on Kant’s concept of a regulatory principle as a standard “with which we can compare ourselves, judging ourselves and thereby improving ourselves, even though we can never reach the standard.” Democratization is a process and progresses by degrees with no predetermined end point for which to aim. We can evaluate how different aspects, processes, and policies for PPIE in research improve or reduce their democratic quality—how far they democratize health research. Hence, democratization is not an absolute category; there can be degrees of democratization, and democratic quality can be improved (or decreased) incrementally by adjusting existing procedures and institutional structures (Stevenson, 2016). PPIE is part of the processes designed to democratize health research; conceptualizing elements of PPIE as a form of deliberative democracy gives us a way to judge and improve practice.

## V. RESEARCH AGENDAS

Conceptualizing the central function of PPIE as a way of democratizing health research provides a starting point for assessing how well PPIE fulfills this function, providing a yardstick against which to judge the legitimacy of decisions and practices, and how to create the conditions for more meaningful deliberation. As Gutmann and Thompson say, “The theory of deliberative democracy offers a useful standard by which to judge actual decision-making as better or worse, to the extent that the reasons for the decisions are mutually justified” (2004, 141). In this section, a broad research agenda is sketched out, pointing to areas and questions that merit further exploration.

### Empirical Research

PPIE in research takes places in a large range of settings and contexts and these specific instances of involvement need to be empirically studied alongside theoretical work to determine appropriate, normatively justifiable, workable criteria for deliberative practices in these areas. As Neblo et al. note, “many of the big advances in our understanding of deliberation are likely to come by carefully aligning normative and empirical inquiries in a way that allows the two to speak to each other in mutually interpretable terms” (2010, 566)

There is now a growing body of empirical research on deliberation in the wider political realm (Thompson, 2008; Elstub, Ercan, and Mendonça 2016), from concerns that deliberative democracy lacked sufficient empirical investigation (Bohham, 1998), Dryzek has now noted an “empirical turn in deliberative democracy.” There has also been a substantial body of research on public participation and public deliberation in the health arena (see Blacksher, 2013, for an overview). Studies have either considered real-world examples to explore deliberative practices (Parkinson, 2006) or constructed deliberative forums as part of research projects (Fishkin and Luskin, 2005).^7^ To date, the majority of studies have focused on deliberative methods in areas such as: health policy (Abelson et al., 2007; Degeling et al., 2015), technology assessment (Abelson et al., 2016), and assessment of the use of medical evidence (Carmen et al., 2015). Only a limited amount of research has focused on using deliberation in applied health research projects specifically, with the exception of De Vries et al.’s (2010) study, for example, on informing policy for the use of surrogate consent to enroll persons with dementia in clinical research. Consequently, there is a need for more research that focuses on how deliberation does, and could, operate specifically at the research project level.

A starting point for this type of research program is to explore how deliberation is carried out in practice to develop a workable concept of deliberation, as Thompson notes, “While claiming (correctly) that deliberative theories share a common core of values, the empirical studies actually adopt diverse concepts of deliberation and examine different consequences under a range of conditions” (2008, 501). Steiner (2008) calls this problem “concept stretching”: deliberation has become used to mean any form of talk; this lack of conceptual clarity has made it hard to draw more widely applicable conclusions from studies. It is important to demark deliberation from other forms of talk and discussion, or deliberation becomes about everything and hence, about nothing. Empirical research can enhance our understanding of how deliberation takes place in different contexts. By seeing how the practice of deliberation fits with existing theory and whether the theory needs to be adjusted, developed, or changed in light of practice, progress can be made on both understanding deliberative practices and developing more nuanced, and practically useful, normative theories and related guidelines. Empirical research needs to move carefully between conceptual definitions and practice, treading the line between imposing abstract concepts on practice or losing any normative and explanatory value by jettisoning them completely. Ways of integrating empirical research and normative concepts have been developed in a number of areas, such as empirical ethics (Molewijk and Frith, 2009). Methodologies such as symbiotic empirical ethics (Frith, 2012), that sees practice as informing theory just as theory informs practice, can contribute to theory development.

### Assessing Deliberative Practice

A key area for investigation is how deliberative processes can give greater legitimacy to decisions. Many authors have discussed conditions that should be met in order for deliberative forums to produce legitimate results (Gutmann and Thompson, 2004; Parkinson, 2006; Mansbridge et al., 2010) and a growing body of research focuses on how deliberation can be assessed in practice. For example, Steiner’s work on assessing the quality of discourse in deliberation by using a quantitative measure, the “Discourse Quality Index” (Steenbergen et al., 2003; Steiner, 2012), and Drysek’s (2009) idea of deliberative capacity, used to evaluate a polity’s deliberative systems. Dryzek puts forward three criteria for how well a system or parts of a system promote effective and meaningful deliberation, what he terms “deliberative capacity.” “Deliberative capacity may be defined as the extent to which a political system possesses structures to host deliberation that is authentic, inclusive, and consequential” (Dryzek, 2009, 1382). First, authenticity refers to deliberation that is non-coercive, relates views of particular groups to more general principles, and is reciprocal—providing arguments in terms that others can accept (Guttman and Thompson, 2004). There has been debate over the types of discursive practices that can be said to be authentic; does it have to be confined to reasoned argument (*pace* Habermas), or, as Dryzek suggests, rhetoric, personal stories, and humor should be included as acceptable forms of discursive practice. What this aspect of legitimacy might look like in health research and how processes are created to facilitate free and open exchange to encourage values-based reasoning (Abelson et al., 2013) need to be explored. Second, inclusivity requires that a range of interests is present in the dialogue, and that particular groups are not, a priori, excluded. This connects back to worries that PPIE is often not sufficiently diverse or inclusive with an insufficient range of voices being heard. Finally, the deliberation must be consequential, it must have an impact on the outcomes. Dryzek states that it need not involve actually making the [policy] decisions but it must affect or influence the decision in some way. This is reflected in the growing movement in the PPIE community that “good” PPIE is more than just dissemination and consultation. Public contributors should take on more active roles and genuinely co-produce research and hence, be involved in making key decisions.

### Research Questions

Exploring PPIE in health research through the lens of deliberative democracy opens up a range of new and productive avenues of investigation. As a starting point for further research, this section concludes with highlighting three research questions that could be usefully explored.^8^ First, what role should diversity play in deliberative forums in health research? The importance of bringing in diverse and broadly non-traditional expertise is often cited as an important justification for PPIE in research. Although this idea of giving a voice to patients and marginalized groups is deemed important, what is left unclear is which diverse perspectives are needed? What range, breadth, and type of expertise do we need to do PPIE well? Do we need “a diversity of population characteristics vs. a diversity of viewpoints?” (Abelson et al., 2013). Second, how the deliberative processes in PPIE in research fit in the wider democratic systems of a society needs to be considered. When assessing the democratizing potential of recent deliberative approaches, people such as Kuyper (2016) and Parkinson and Mansbridge (2012) have taken a systems approach to deliberative democracy, arguing that no single institution or forum can foster deliberative democracy alone; this needs to be done across a system. Hence, when assessing the democratizing potential of deliberation in health research, its place in the wider system and how it fits with other institutions and deliberative spaces need to be considered. Third, who should be responsible for putting these processes in place and ensuring that health research fosters democratic processes? One obvious candidate is the funders of health research; the NIHR in the United Kingdom has been a major driver for PPIE, and funders are well placed to put conditions and requirements on health research practice. Who has responsibility for promoting the democratizing of health research and the duties and obligations this entails and the type and limit of these need to be examined.

Considering these types of question and how criteria for good deliberation can be used to assess PPIE can provide a framework for developing research programs to explore deliberative processes in health research.

## VI. CONCLUSIONS

It has been argued that one of the key functions of PPIE is to make health research more democratic. If what is expected from PPIE practices is made explicit, then activities can be developed that further these goals and minimize conflicts that can result from different perceptions of the rationale for PPIE (Dean, 2017; Wilson et al., 2018). It can help us both to construct mechanisms that foster democratic practices and to determine legitimacy and accountability by providing tools and resources to assess these decision-making processes. The aim of democratizing research is both an ongoing process but also a relative one. Research probably will never be fully democratic—if indeed any area is—and it does not need to be. The goal is to make it more democratic and to determine the appropriate operational parameters of this. Specific instances of PPIE in health research need to be empirically studied to develop theories of what are appropriate, normatively justifiable, and workable criteria for deliberative practices in these areas. By doing this, we can improve practice by having a firmer basis for normative assessment of PPIE in health research, giving us greater conceptual clarity over what PPIE in research is trying to achieve, and providing criteria for determining if PPIE initiatives meet these ends.

## Footnotes

1 Involve has now passed its functions over to a new organisation called the NIHR Centre for Engagement and Dissemination.

2 PPIE in research can lay the groundwork for improving health care and services for disadvantaged groups; see Wallerstein, 2006; Engel et al., 2018. It can also have personal benefits for public contributors, developing life and employment skills and empowering individuals and communities (Popay and Porroche-Escudero, 2018).

3 It can be argued that deliberative democracy can enhance the quality of participatory democracy (see Dryzek and Lo [2015]), and deliberative and participatory democracy are not antithetical conceptions of democracy, as has often been argued (Curato et al., 2017).

4 Debates over what form of deliberative democracy might best characterize PPIE in research need to take place; there is no space in this paper to consider this in the depth that would be required to make headway. A starting point might be making a distinction between types and levels of deliberation, macro deliberation in civil society and micro deliberation in small-scale forums. As Henricks notes: “In order to achieve the communicative conditions of micro deliberation, participant numbers tend to be small—hence, the ‘micro’ label. In this respect, micro accounts tend to privilege the reasoning dimension of deliberation over participation” (2009, 176). Thus, PPIE in research might productively be seen as a form of micro deliberation.

5 This is a broad overview; there is no space in this paper to do justice to the diverse nature of deliberative democratic theory and the debates between the different perspectives (Elster, 1998; Chambers, 2003).

6 There is a significant literature debating the merits of deliberative democracy, see Gutmann and Thompson (2004) and Curato et al. (2017) for a rebuttal of some of the critiques outlined here.

7 For examples of Fishkin’s more recent work at the Center for Deliberative Democracy, see https://cdd.stanford.edu\.

8 These are just initial suggestions for future work and are by no means exhaustive of the kinds of questions or areas that could be investigated.
